# Students as co-designers in health professional education: a scoping review

**DOI:** 10.1186/s12909-025-07110-0

**Published:** 2025-05-03

**Authors:** Élodie Ambrosetti, Cyrille Gaudin, Simon Flandin, Germain Poizat

**Affiliations:** 1 University of Geneva, Faculty of Psychology and Educational Sciences, and University of Applied Sciences and Arts Western Switzerland, Geneva, Switzerland; 2 University of Limoges, National Higher Institute for Teaching and Education, Limoges, France; 3 University of Geneva, Faculty of Psychology and Educational Sciences, Geneva, Switzerland

**Keywords:** Student-staff partnership, Co-design, Education, Health professions, Literature review

## Abstract

**Background:**

Over the last thirteen years, there has been a notable increase in both research and practice related to student-staff partnerships in higher education. However, within health professional education (HPE), studies on these partnerships remain limited and often rely on broader higher education frameworks. Existing research primarily focuses on role dynamics and relational aspects rather than on structured co-design processes, where students actively contribute to shaping educational content, assessments, or curricula. Building upon previous work, this study specifically examines co-design as a distinct dimension of student-staff partnerships in HPE, an area that has not been thoroughly addressed in recent literature reviews.

**Methods:**

In accordance with the PRISMA-ScR 2018 statement, we performed searches in online databases—Cochrane, Ovid, PubMed, ScienceDirect, and Scopus—for original articles published in English from 2010 to 2023. These articles needed to describe empirical studies focused on co-designed training programs in health professions. We then conducted a qualitative and descriptive analysis of the selected articles to examine how the principle of students as co-designers is portrayed and investigated in health professional education.

**Results:**

The search (title, abstract, keywords) identified 703 potentially relevant abstracts addressing co-design in healthcare education. Screening of these abstracts narrowed it down to 84 articles. Further evaluation of these full articles resulted in a final sample of 20 articles that met the inclusion criteria. We analyzed the content of these 20 articles using the following categories: basic characteristics (year of publication, country, professional domain, educational grade, topic of the training), co-design characteristics (context and initiative, framework and definition, purposes, stakeholders, process), and study characteristics (aim, research framework, population, data collection and analysis, key findings). Our analysis revealed that co-design in HPE lacks standardized frameworks and rigorous empirical evaluation. Many studies emphasize student contributions but do not provide detailed methodological guidance on how co-design is structured, implemented, or assessed. Additionally, findings indicate that most studies focus on undergraduate education, with postgraduate applications remaining underexplored.

**Conclusions:**

This review underscores co-design as an emerging yet underdeveloped approach in health professional education. While its potential benefits—such as enhancing student engagement, fostering innovation, and improving training relevance—are widely acknowledged, the field lacks structured methodologies and theoretical grounding. Future research should focus on developing clear frameworks, assessing co-design’s long-term impact on learning outcomes, and differentiating it from broader collaborative approaches. Strengthening methodological rigor and empirical validation will be essential for positioning co-design as a sustainable and evidence-based practice in health professions education.

**Supplementary Information:**

The online version contains supplementary material available at 10.1186/s12909-025-07110-0.

## Background

This article presents a review of student–teacher co-design in health professional education, defining it as (i) collaborative engagement between students and teachers in creating, modifying, or improving learning, teaching, and curricula, and (ii) a subset of student-staff partnerships in health education. This section provides an overview and rationale for our review.

### Student-staff partnership in higher education

Over the last ten years, we have seen an increase in practices and research on *student-staff partnership* in higher education (e.g., [11, 23, 28, 43]). A wide variety of terms have been used to describe this partnership in *teaching, learning and research*: students as co-researchers (e.g., [42, 69]), students as co-inquirers (e.g., [7, 32, 89]), students as co-developers (e.g., [33, 48]), students as co-producers (e.g., [46, 70, 92]), students as co-authors (e.g., [45]), students as change agents (e.g., [27, 40, 82]), students as co-creators (e.g., [12, 17, 22, 36]), students as co-designers (e.g., [30, 34, 91]), students as co-directors (e.g., [23, 68, 74, 77]). This variation in terminology poses a methodological challenge for obtaining generalizable findings, but this diversity of ways of naming this kind of educational practice is also important because it reflects specific disciplinary and cultural characteristics [21, 41].

This results in various models, frameworks, and typologies, some of which are particularly relevant for explaining what is at stake, as illustrated below. Dunne and Zandstra [27] developed a *theoretical framework for engaging students as change agents*. Their approach is designed around two key dimensions: (a) the extent to which any activity is led by students or led by the institution,and (b) the extent to which any activity is premised on active engagement by students in change, or is based on more passive forms of representation. The four elements of the model are students as evaluators, students as participants, students as partners and students as change agents. Dunne [26] developed another framework that distinguishes between student engagement in critical thinking (focused on analysis—‘breaking down ideas’) and student engagement in design thinking (focused on improvement—‘building up ideas’). This framework provides a valuable addition to the repertoire of understandings regarding student engagement.

Likewise, Healey et al. [43, 44] created *a conceptual model for partnership in learning and teaching*. The model distinguishes two spectrums of engaging students as partners. The first spectrum extends from learning, teaching and research (through learning, teaching assessment,subject-based research and inquiry) to quality enhancement of learning and teaching practice and policy (through teaching and learning scholarship; curriculum design and pedagogical consultancy). The second one extends from co-learning, co-designing and co-developing (through learning, teaching assessment; curriculum design and pedagogical consultancy) to co-researching and co-inquiring (through subject-based research and inquiry; teaching and learning scholarship). However, the authors state these distinctions are blurred, and the inter-relationships between different aspects are complex and diverse when put into practice.

Eventually, Bovill et al. [13] identified *four roles* students often take on in *co-creating learning and teaching*: (a) consultant (sharing and discussing valuable perspectives on learning and teaching),(b) co-researcher (collaborating meaningfully with staff on teaching and learning research or subject-based research); (c) pedagogical co-designer (sharing responsibility for designing learning, teaching and assessment); and (d) representative (student voices contributing to decisions in a range of university settings). Bovill et al. [13] note these roles are not mutually exclusive,indeed, significant overlap may occur. This model has been enriched with the *participation matrix framework*, which explores roles within student-staff partnerships in higher education [9, 56]. The framework helps consider which students and staff should be partners, when and in what ways. For example, students might be informed, consulted, involved, partners, or leaders of the work. This framework has been complemented by the *co-creation of a learning and teaching typology* [10], which is a practical resource intended to support students and staff to reflect on their planned and current practice and discuss it, to be able to identify what particular kind of co-creation they are doing or planning to do. The typology includes a list of co-creation variables, presented in the form of questions (e.g., who initiates co-creation?), followed with different responses to these questions (e.g., staff-led or student-led or both staff and student-led), which illustrate the different possible co-creation types. The authors see the typology as having the potential to becoming a planning tool, a reflective tool, and a mapping tool.

Despite these useful research attempts to map the field, the literature on student-staff partnership in higher education remains fragmented. For example, in an attempt to disentangle the terminology, Martens et al. [65] identified the three most frequently used terms related to the design of learning and teaching: *design-based research* (DBR), *participatory design* (PD), and *co-creation*. The results of their study show much overlap between these terms. The similarity between DBR, PD and co-creation lies in valuing the input of students as stakeholders in the educational design process. However, when trying to differentiate terms, key differences lie in the level of student participation during the design process and regarding the focus on educational theory. Depending on the approach to design in learning and teaching, students increasingly become the central actors (from user, to tester, informant and design partner), while the focus on educational theory decreases. Martens et al. [65] therefore point out “it is important the level of student participation be aligned with the purpose of the approach” (p. 1205).

In addition to this finding, the systematic literature review by Mercer-Mapstone et al. [73] identified four main themes depicting how *students as partners* practices in higher education are presented in the academic literature: (a) the importance of reciprocity in partnership,(b) the need to make space in the literature for sharing the (equal) realities of partnership; (c) a focus on partnership activities that are small-scale, at undergraduate level, extracurricular, and focused on teaching and learning enhancements; and (d) the need to move toward inclusive, partnered learning communities in higher education. Nevertheless, as Barradell and Bell [5] point out, “Students as partners is a movement which is gaining momentum in higher education, yet disciplinary perspectives are underexplored” (p. 513).

### Student-staff partnership in health professional education

The health professional education literature on student-staff partnership is scarce and therefore relies mostly on higher education literature. Targeting medical educators, Könings et al. [57] relied on higher education literature of co-creation to develop the *framework of stakeholder involvement in co-creation*, which depicts the effects of different stakeholders’ active involvement in the educational design process (e.g., learners’ involvement leads to improved learning processes,teachers benefit from co-creation, as dialogues with learners improve teaching practices and foster their own professional development). Könings et al. [57] also describe potential challenges and barriers to implementing co-creation in practice from learners’ perspective (e.g., power relationships), teachers (e.g., giving up control), and institutions (e.g., lack of support). In addition, most articles provide practical tips for health professions educators interested in implementing student-staff partnership initiatives at their own institutions (e.g., [47, 57, 83]).

To our knowledge, three literature reviews have been conducted on student-staff partnership in health professional education. The first one focused on nursing and midwifery education, and extended the partnership to service users or carers, and is thus limited to siloed health professions [76]. The second one encompassed all health professions, but explored only qualitative studies on partnering with students [5]. The third one studied specifically how co-designing education with students is practised across formal curricula in health professions programs drawing on qualitative, quantitative and mixed methods research [1], which positions it as a significant earlier work in relation to ours.

### Research questions

In our view, addressing the fragmented nature of this literature requires producing reviews that clearly define (i) the scope studied within the broad field of “student-staff partnership” and (ii) the specific professional and educational contexts under consideration. Furthermore, the foremost need in this literature is for studies that provide both conceptual clarity and empirical evidence on the diverse dimensions of student-staff partnerships.

Building on the insights from Abbonizio et al. [1], our study seeks to delve into a specific dimension of “students as partners” that represents a research gap: co-design. While “partnership” and “students as partners” often focus on roles and the social relationships between participants, “co-design” specifically pertains to the act of “making”—that is, co-design as a practice. Following Bovill et al. [13], we define co-design as a role where students actively engage as pedagogical co-designers in the creation of learning, teaching, and curricula. We selected the scope of co-design as a priority because it encapsulates the essence of student-partnership ideals, representing the highest degree of student engagement.

To investigate this focus, we conducted a systematic inquiry guided by two research questions:

- Research question 1 (RQ1): How is the concept of students as co-designers depicted in the literature, and what purposes does it serve in the context of health professional education?
- Research question 2 (RQ2): How has the role of students as co-designers been studied in health professional education research, and what outcomes have been reported?

## Method

### Data collection

**Fig. 1:**
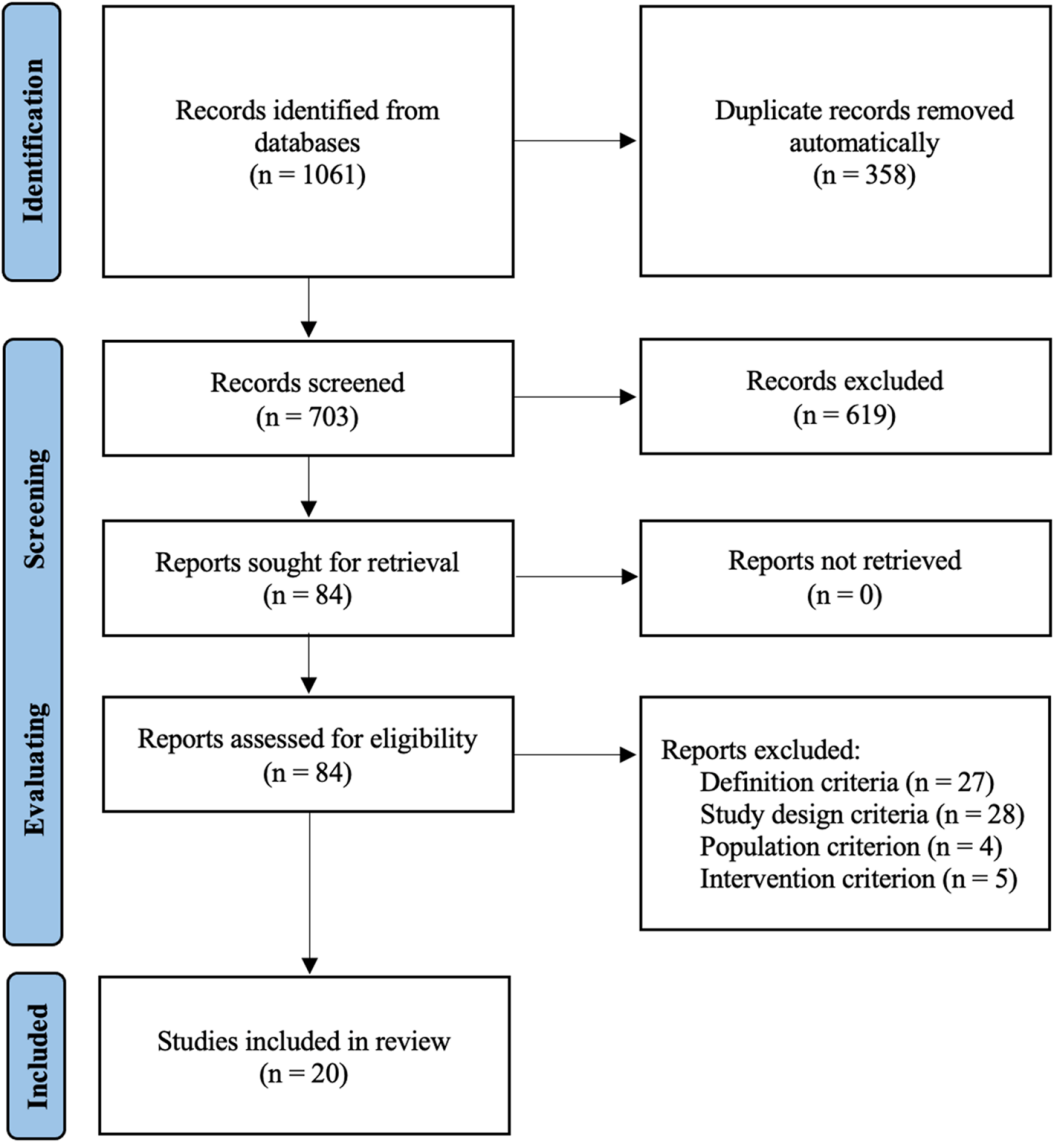
Flow diagram of the data collection process – PRISMA 2020 [79]

We followed the Preferred Reporting Items for Systematic reviews and Meta-Analyses extension for Scoping Reviews (PRISMA-ScR) 2018 statement [86] to identify relevant studies for this scoping review. The data collection process was achieved in three steps: identification, screening and evaluating (Fig. 1).

### Identification

This step consisted firstly of establishing a search query: (participatory design OR co-design OR co-creation OR co-construction OR co-production OR co-development OR collaborative design) AND (learner OR student OR trainee). After reading the first identified articles, this search query was supplemented by a new one: (student staff partnership OR student staff collaboration OR student faculty collaboration OR student faculty partnership OR students as partners). In addition to keywords, the search query integrated the selection level of the publications (title OR keywords OR abstract) and general criteria: period (must have been published between 2010 and 2023); type of publication (must be a peer-reviewed article); language (must be in English); professional fields (must be in the field of health professions).

Next, the search query (refer to Table A1 in the additional files for further information) was applied in five online databases: Cochrane, Ovid, PubMed, ScienceDirect and Scopus. These were chosen because their search tools allow the selection of articles to be restricted to the field of health professions. Eventually, 703 potentially relevant articles were identified on April 4 th, 2024 (Table 1). The references retrieved were exported to EndNote version X9, and the duplicates were removed automatically.

**Table 1:** Details of the articles search

Online databases	Number of articles retrieved on their title-abstract-keywords
Cochrane	22
Ovid	26
PubMed	522
ScienceDirect	67
Scopus	424
Total	703(358)

*The number in parentheses refers to the removed duplicates*

### Screening

This step firstly consisted of establishing the inclusion and exclusion criteria (Table 2), and secondly to apply these criteria to the abstracts. In addition to general screening criteria (criteria a, b, c, and d), four supplementary criteria were specified: definition (criteria e, f, and g), study design (criteria h and i), population (criterion j) and intervention (criterion k).

**Table 2:** Inclusion and exclusion criteria

	**Inclusion criteria**	**Exclusion criteria**
General criteria	**a.** Must have been published between 2010 and 2023	**a.** Publication before 2010 and after 2023
	**b.** Must be peer-reviewed	**b.** Publications that are not peer-reviewed
	**c.** Must be in English language	**c.** Publications in other languages than English
	**d.** Must be in the field of health professions	**d.** Publications on professions other than health
Definition	**e.** Co-design consists of students and faculty staff working together in a structured space towards educational and training purposes, with potential other stakeholders (e.g., healthcare professionals, patients, design professionals)	**e.** Co-design as participatory research, dialogical communities, peer-learning, or knowledge and value co-creation
	**f.** Students significantly involved in co-design process (e.g., ideation, decision, making) resulting in concrete changes (e.g., course, scenario, tool, curriculum) **g.** The beneficiaries of co-design process are the co-designer’s students, their peers or the following student classes	**f.** Student participation in co-design is limited to providing information (e.g., responding to a survey). Student solely as a tester (e.g., giving feedback) or the final user of a product co-designed by other stakeholders **g.** The co-design process beneficiaries are the students’ partners (e.g., faculty staff, teachers, professionals, patients and care receivers) or a third party
Study design	**h.** Must be an empirical study **i.** Co-design is one of the main topics and is described and analyzed precisely	**h.** Literature reviews, commentary papers, conceptual articles, study protocols **i.** Co-design is a peripheral topic
Population	**j.** Student as undergraduate student (entry-level educational context) or professional in training (postgraduate continuing study once qualified to practice)	**j.** Student as a pupil or a patient
Intervention	**k.** Co-design in health professional education and training (e.g., medicine, nursing)	**k.** Co-design of preventive action in healthcare

Firstly, regarding the *definition* (criteria e, f, and g), studies were included if the co-design consisted of students and partners (e.g., faculty staff, teachers, professionals and patients) working together in a structured space and time towards attaining educational and training purposes. Studies that considered co-design as participatory research (e.g., [2, 75]), dialogical communities (e.g., [8, 24]), peer-learning (e.g., [16, 59]), or knowledge and value co-creation (e.g., [31, 50]) were excluded. In addition, studies were included if students were significantly involved in the co-design process (e.g., ideation and decision-making) resulting in concrete changes (e.g., course, scenario, tool and curriculum). Studies where student participation in co-design is limited to providing information (e.g., responding to a survey: [55, 63]), only be a tester (e.g., giving feedback: [53, 80]), or one of the final users of a product co-designed by other stakeholders (e.g., [3, 61]) were excluded. Finally, even if there are benefits for students to participating in the co-design process itself [76], studies were included only if the designated beneficiaries of co-design process were the co-designer’ students, their peers or the following student classes. Studies were excluded if the designated beneficiaries of the co-design process were the students'partners (e.g., faculty staff, teachers, professionals, patients, or care receivers) or a third party (e.g., [49, 78]).

Secondly, regarding the *study design* (criteria h and i), only empirical studies were included. Excluded were literature reviews (e.g., [5]), commentary papers (e.g., [47]), conceptual articles (e.g., [83]) and study protocols (e.g., [87]). In addition, studies were included if the co-design was one of the main topics and precisely described and analyzed. If the co-design was a peripheral topic, studies were excluded (e.g., [4, 19]).

Thirdly, regarding the *population* (criterion j), included were studies where the student was assimilated to an undergraduate student (entry-level educational context) or a professional in training (postgraduate continuing study once qualified to practice). Studies where students were assimilated to secondary school aged pupils (e.g., [20]) or patients (e.g., [90]) were excluded.

Fourthly, regarding the *intervention* (criterion k), studies addressing co-design in health professional education and training (e.g., medicine, nursing) were included. Studies addressing co-design of preventive action in healthcare (e.g., adolescents’ perspectives on substance use prevention) were excluded (e.g., [20]).

Based on these criteria, first author screened the titles and abstracts of the 703 identified studies. Independently, the other three authors each screened a third of these identified studies to achieve a double screening of the corpus. Discordant screenings were resolved by discussion until consensus was reached. These exchanges also made it possible to refine the inclusion and exclusion criteria. In the end, 84 articles were retained for the evaluating stage.

### Evaluating

As in the screening stage, first author evaluated the 84 full texts based on the inclusion and exclusion criteria. Independently, the other three authors evaluated a third of these full texts to double evaluate the corpus. Discordant evaluations were resolved by discussion until consensus was reached. In the end, 20 articles were included in the review.

### Data coding and analysis

An Excel spreadsheet was used for systematizing the data coding and analysis process. The authors collaboratively created a set of data categories and subcategories, presented in Table 3, in order to methodically identify in the articles (a) basic components and (b) specific components that related to research questions.

**Table 3:** Data categories and subcategories

Categories	Subcategories
Basic characteristics	Year of publication, country, professional domain, educational grade, topic of the co-design activity
Co-design characteristics **(RQ1)**	Context and initiative, framework and definition, purposes, stakeholders, process
Study characteristics** (RQ2)**	Aim, research framework, population, data collection and analysis, key findings

Finally, the first author coded all included articles. Independently, the other three authors coded one-third of the included article to achieve a double coding of the whole corpus. Disagreements were resolved by discussion until consensus was reached. Outcomes for each subcategory were synthesized and tabulated or visually displayed, including the 20 studies each time.

### Findings

This section presents a descriptive analysis of the 20 studies included in this scoping review. It is structured into three sections: basic characteristics, co-design characteristics (RQ1) and study characteristics (RQ2).

### Basic characteristics

From the studies analyzed, we retrieved basic characteristics such as publication frequency, geographical distribution, professional domains covered, and educational grades (Table 4).

**Table 4:** Basic characteristics of the co-designed training programs

Characteristic	Details
Publication Frequency (2010–2023)	2017 (4), 2018 (2), 2019 (4), 2020 (3), 2021 (2), 2022 (4), 2023 (1)
Geographical Distribution	USA (5), UK (2), Canada (2), Netherlands (2), Germany (2), Norway (2), France (2), Denmark (1), Australia (1), New Zealand (1)
Professional Domains Covered	Physicians (10), Various Others (10): Nurses, Veterinarians, Radiographers, Occupational Therapists, Pharmacists, Midwives
Educational Grade	Undergraduate (17), Postgraduate (2), Both (1)

*The number in parentheses refers to the number of studies*

The broad majority of studies (*n* = 17) examine students as co-designers in health professional education at undergraduate level. Two studies address it at postgraduate level [62, 67] and one study addresses it simultaneously at both levels [72]. This result reflects an unbalanced distribution of the levels of training examined. As a matter of fact, undergraduate health professional programmes are (i) basically far more numerous than postgraduate programmes, and (ii) more structured and standardized, making them more suitable for co-design interventions. On the opposite, postgraduate programs are typically shorter, more intense, and competency-driven, leaving little room for iterative co-design processes.

The topic of the co-design activity (central theme, subject matter) was also retrieved (see Table 5, at the end of the Results section).

**Table 5:** Summary of the studies characteristics

Study Characteristics	Category	Details *(The number in parentheses refers to the number of studies)*	Studies
Topics	Soft Skills Topics	Leadership, Communication, Racial Tensions, Reducing Nurse Burn-out, Interprofessional Collaboration, Teamwork, Confidence and Awareness	Ha and Pepin [37], Martin et al. [67], Brook et al. [15], Behrend et al. [6], Kayser et al. [52], Laugaland et al. [58]
	Hard Skills Topics	Pathology, Pain Management, Radiotherapy Planning, Neurorehabilitation, Newborn Resuscitation	Kenwright et al. [54], Bradshaw et al. [14], Chamunyonga et al. [18], MacKenzie et al. [62], Ljungblad et al. [60], Eveillard et al. [29], Greenhouse et al. [35]
	Not Specified Topics	Various topics not explicitly stated or not tied to a specific curriculum or evaluation purpose	Martens et al. [66, 72], Milles et al. [74], Scott et al. [84], Tavernier and Wolfe [85], Cosker et al. [25], Harrison et al. [39]
Main Approaches	Approaches to (Co-)Design	Participatory Action Research (PAR)	Kenwright et al. [54]
		Participatory Design Principle	Harrison et al. [39]
		Students as Partners	Chamunyonga et al. [18], Cosker et al. [25]
		Students as Module Co-directors	Milles et al. [74]
		Student Integration Model (SIM)	Tavernier and Wolfe [85]
		Co-creation Process	Laugaland et al. [58]
		Human-Centred Design Principles	Scott et al. [84]
	Pedagogical Approaches	12-domain HSS Framework	Greenhouse et al. [35]
		Kern Model of Medical Curricular Development	Bradshaw et al. [14], Kayser et al. [52]
		Problem-Based Learning (PBL)	Martens et al. [66]
		Critical Pedagogy Framework	Martin et al. [67]
		Best Practice Simulation Standards	MacKenzie et al. [62]
		Studies Without Clear Framework	Ha and Pepin [37], Brook et al. [15], Behrend et al. [6], Meeuwissen et al. [72], Eveillard et al. [29], Ljungblad et al. [60]
Purposes of Co-Design	Courses Development	Content Course	Eveillard et al. [29, 65], Ljungblad et al. [60]
		Interprofessional Courses	Behrend et al. [6]
		Pain-related Content	Bradshaw et al. [14]
		Learning Activities	Ha and Pepin [37]
		Course Revision	Kenwright et al. [54]
	Simulation Development	Simulation	MacKenzie et al. [62]
		Simulation Scenarios	Martin et al. [67], Kayser et al. [52]
	Assessment	Assessment Tasks	Chamunyonga et al. [18]
		Summative Assessment Culture	Harrison et al. [39]
		Objective Structured Clinical Examination (OSCE)	Cosker et al. [25]
	Program construction	Governance	Meeuwissen et al. [72]
		Curriculum	Milles et al. [74], Scott et al. [84], Tavernier and Wolfe [85]
	Educational creation or intervention	Intervention	Brook et al. [15], Greenhouse et al. [35]
		Digital Resource Creation	Laugaland et al. [58]
Aims of Co-Design Analysis	Description	Experience	Ha and Pepin [37], Brook et al. [15], Behrend et al. [6], Kayser et al. [52], Laugaland et al. [58]
		Perception	Eveillard et al. [29] (about the co-creation format) Martens et al. [66] (conceptions of student participation) Martin et al. [67] (challenges and frustrations) Milles et al. [74] (role, functions and effects of the student module co-directors) Behrend et al. [6] (process and results of interprofessional course development)
		Types of Learning	Kenwright et al. [54]
		Concepts, Activities and Practices	Milles et al. [74]
		Engagement Factors	Behrend et al. [6] (what facilitated students’ involvement) Greenhouse et al. [35] (what authentically engages students) Meeuwissen et al. [72] (what factors render student participation in medical and veterinary education governance successful or not)
		Design Guide	MacKenzie et al. [62], Cosker et al. [25], Ljungblad et al. [60]
	Evaluation	Perception	Cosker et al. [25] (perceived effectiveness) Kayser et al. [52] (of learning objectives)
		Effects	Eveillard et al. [29] (on learning outcomes) Kenwright et al. [54] (motivation, self-efficacy) Martin et al. [67] (self-reflection, effectiveness at getting into another person’s experience) Behrend et al. [6] (how this affected student’s professional development) MacKenzie et al. [62] (impact on student learning) Tavernier and Wolfe [85] (the perceived impact on students, faculty and program improvement)
		Process	Martin et al. [67] (conduciveness to learning, relevance and applicability to practice and training, realism in the SP’s portrayal of the patient)
		Engagement factors	Brook et al. [15] (acceptability of co-production as a process, and the feasibility and value of co-production) Meeuwissen et al. [72] (the perceived value of student participation) Scott et al. [84] (the Ed Reps program use and acceptability)

The choice of the topic is linked to (a) addressing a need in the field or a professional issue (often underestimated in the current curriculum) – for example, tasks and assessments must mimic the complexity of ‘real-world’ treatment planning scenarios students may encounter in professional life be implemented [18], and/or (b) a recommendation from healthcare experts – for example, more emphasis on interprofessional education as a key approach [6], and/or (c) a finding from the scientific literature – for example, a paucity of evidence relating to the explicit participatory nature of work to design interventions meant to increase nurse retention [15]. Furthermore, topics are chosen by researchers and/or trainers arbitrarily and for convenience [67] or provide some freedom of choice for students in terms of the topic-related content or the content delivery method [6, 18, 54, 67]. Finally, no study mentions that students were involved in choosing the topic of the course in the co-design process. For example, in the study by MacKenzie et al. [62] on the co-construction of a simulation, the topic, content and teaching method were defined by the trainer and only the learning objectives were negotiated with students.

### Co-design characteristics

This section aims to answer the first research question: How is the concept of students as co-designers depicted in the literature, and what purposes does it serve in the context of health professional education?

### Co-design context and initiative

In nearly all studies, a commitment to improving teaching and learning is expressed through students’ involvement in co-design. This intention is held by one or more elements summarized in Table 6.

**Table 6:** Co-design’s drivers in student training

Driver	Purpose	References
Literature in Higher Education	Highlights international efforts to engage students in designing their education, noting benefits such as increased success, employability, engagement, and improved assessment	Ha and Pepin [37], Cosker et al. [25], Greenhouse et al. [35], Tavernier and Wolfe [85]
Quality Assurance Approaches	Includes programs aimed at recognizing excellence in medical, dental, and veterinary education internationally, and efforts to improve higher education quality in the UK	ASPIRE program (as referenced by Kayser et al. [52], Martens et al. [66], Meeuwissen et al. [72], Milles et al. [74]); Quality Assurance Agency for Higher Education (as referenced by Brook et al. [15])
Institutional Initiatives	Involves student co-design to improve teaching and learning outcomes within institutions	Behrend et al. [6], Bradshaw et al. [14], Harrison et al. [39], Ljungblad et al. [60], Scott et al. [84]
Research Projects	Focus on co-design processes in specific studies to explore and validate educational methods and practices	Chamunyonga et al. [18], Kenwright et al. [54], Laugaland et al. [58], MacKenzie et al. [62], Martin et al. [67]

Almost all described training programs were studied during their initial implementation for testing, assessment, or as a proof-of-concept. Consequently, there is limited information about the potential of long-term, established co-designed programs.

### Co-Design Framework and Definition

Across all the studies reviewed, a variety of adjectives were used to describe co-design. The term most commonly used is student-staff (or faculty) partnership (or collaboration), which appears in six studies [14, 25, 66, 72, 84, 85]. This is closely followed by co-creation, which is found in five studies [29, 35, 54, 58, 60]. The qualifier *co-construction* is used in three studies [37, 62, 67], two of which pertain to the medical simulation device (the study by Martin et al. [67] being inspired by the device and the results of MacKenzie et al. [62]. The other qualifiers (co-production for Brook et al. [15], students as partners for [15, 18], student engagement and co-directors for [74], co-development for [52], student participation for [6] and participatory design for [39] are mentioned in only one study.

Seven studies draw on a referenced definition of the qualifier mobilised [15, 18, 29, 35, 37, 54, 65]. For example, Martens et al. [65] define the qualifier student staff partnership according to the acceptance of Cook-Sather et al. [23]. Nine studies use references that themselves sometimes mobilise other terms [6, 14, 25, 39, 52, 58, 67, 72, 74]. For example, Bradshaw et al. [14] use the term student-faculty collaboration and draw on a reference dealing with “student-led” collaboration. Four articles out of 20 do not provide any definition or reference directly relating to co-design [60, 62, 84, 85]. Several studies (e.g., [35, 39, 54, 74, 84]) use several qualifiers indiscriminately (e.g., co-design, co-creation, co-development), leading to a notional blurring of co-design in these studies.

'Co-construction'is frequently associated with constructivism and collaborative learning paradigms. These terms primarily originate from the fields of Higher Education, Medical Education, Psychology, and Policy Sciences. Their definitions, often building upon previous studies, illustrate an academic lineage of terms, reflect growing interest, and contribute to advancing our understanding of training co-design. Therefore, promoting conceptual clarity is crucial, as the absence of direct definitions can lead to ambiguity in the field.

Studies present different definitions of co-design, but also different co-design frameworks (See Table 5).

Unsurprisingly, deriving lessons from the five studies that lack a clearly defined co-design framework is challenging. In contrast, studies that precisely present their frameworks facilitate a clearer understanding and analysis. In the study by Kenwright et al. [54], the Participatory Action Research (PAR) framework offers a methodology for the co-design of revision courses. The authors state that “the focus of PAR is seeking to provide opportunities for researchers and participants to work together at all stages of the study through a cooperative, iterative process of research and action, with power to make decisions which are shared equally among the partners in the collaboration” (p. 652). In the same vein, the co-design methodology deployed by Harrison et al. [39] is borrowed from participatory design principles. They state that “to aid a much closer collaboration between students, teachers and instructional designers, the Combination-Of-Perspectives (COOP) model has been proposed as a way of visualizing the different stakeholders involved. This process of incorporating multiple stakeholders’ perceptions when (re)designing a learning environment is usually referred to as participatory design” (pp. 2–3).

### Co-design purposes

In studies, the purposes of co-design (the intended outcome) are manifold but can be grouped into five categories (see Table 5): (a) courses development; (b) simulations development; (c) assessment; (d) program construction; and (e) educational creation or intervention.

### Co-design stakeholders

In virtually all studies (*n* = 19), students co-designed in collaboration with faculty teachers, except in the study by Brook et al. [15], where a faculty researcher took on the role of the co-design group’s facilitator. The authors distinguish between the members of the co-production group and the facilitator, who is then seen as a guide of the process. Moreover, the researcher’s place and participation in the co-design process are rarely described, as in the study by Ha and Pepin [37]. This may therefore prove difficult to distinguish from those of the faculty staff [14]. Other participants may also emerge such as standardized patients [67], registered midwives [60] or recently graduated young professionals [15].

This option of integrating other types of stakeholders might be linked to the purpose of co-design and the training topic. For example, Brook et al. [15] include an early career nurse as part of their intervention on current nurse workforce issues. On the simulation side, Martin et al. [67] involve the standardized patient in the design of a scenario based on the development of the soft skills needed to care for a patient. It also turns out that the number of students integrated into the group could be the subject of some consideration, as noted in the study by Harrison et al. [39]. The authors suggest that increasing the number of students relative to teachers would help alleviate the power asymmetry that may exist in a collaboration between students and teachers, thereby reducing the hierarchical dynamics within the group. Finally, student experience (years of study) was found to be a selection criterion in ten studies. Kenwright et al. [54], Behrend et al. [6], Chamunyonga et al. [18] and Martin et al. [67] specify the need to include students who already have experience in the academic program, while Scott et al. [84] stress the benefit of involving students at an early stage of their academic program in the co-design of the curriculum in order to encourage longitudinal monitoring of it.

### Co-design process

Studies provide information on three components of the co-design process: (a) temporality, i.e., the time needed for the different co-design stages and for the process as a whole; (b) organization understood as the different phases of co-design and their sequencing; and (c) the content and tasks developed at each stage of the co-design process (refer to Table A2 in the additional files for further information).

Nine studies provide a detailed description of the sequence in terms of temporality, organization and tasks carried out [14, 18, 25, 37, 39, 58, 67, 84, 85]. Some studies offer less detailed or vague descriptions of one or more co-design components, often due to editorial constraints imposed by journal guidelines, which limit the extent to which authors can describe the developed system. Additionally, the aim of the studies may not focus on analyzing the co-design process itself, as seen in Martens et al. [66], where the objective is to examine trainers’ perceptions and preconceptions about including students in course design.

In addition, most studies present (a) a rather imprecise description of the tasks assigned to participants, often confined to a form of discussion, and (b) a degree of diversity and openness in the way co-design sessions are conducted (e.g., number of meetings and timing, tasks carried out and student involvement). Only six studies explicitly detail their methods for constructing co-design (Table 7).

**Table 7:** Methods used to construct co-design in studies

Co-Design Method	Study
Action Research Cycle Design	Kenwright et al. [54]
Experience Based Co-Design (EBCD)	Brook et al. [15]
Kern Model of Medical Curricular Development	Bradshaw et al. [14], Kayser et al. [52]
COmbination-Of-Perspectives (COOP) Model	Harrison et al. [39]
Co-creation Process	Laugaland et al. [58]

Six studies [18, 25, 37, 39, 62, 67] include a preparation phase to encourage participants’ involvement in co-design activities. It takes three forms, (a) preparation for the training device that incorporates co-design: e.g., MacKenzie et al. [62] prepare students for simulation, not co-design,(b) preparation for the purpose of co-design: e.g., Harrison et al. [39] deliver a short presentation on the assessment problem,and (c) preparation for the co-design process: e.g., Chamunyonga et al. [18] specify the involvement of the university coordinator for key information on the value of the partnership approaches.

Ha and Pepin [37] stress the importance of preparing students for the design process. Their study uses the last two formats mentioned (b and c). Participants are prepared not only for the purpose of co-design by providing them with a definition of the topic drawn from a literature review, but also for the co-design process, by informing them about this process framework and challenges during the first meeting (e.g., specifying how the group is expected to function in terms of motivation, power dynamics, respect and the right to speak).

### Study characteristics

This section is intended to answer the second research question: How has the role of students as co-designers been studied in health professional education research, and what outcomes have been reported?

### Aims of the studies

The set of aims described in the 20 studies highlights two trends in the way co-design is approached. In some studies, co-design is understood as a method, i.e., used as a means of designing a training content or device on a specific topic that is itself the object of the study [14, 18, 35, 39, 58, 60]. For example, Chamunyonga et al. [6, 15, 18, 25, 29, 37, 52, 54, 62, 66, 67, 72, 74, 84, 85]. For example, Ha and Pepin [37] seek to describe participant experience during the co-design process. The purpose of co-design (clinical nursing leadership) then becomes a pretext for setting up the co-design process (and does not appear in the study results).

In the studies, co-design is analyzed in the form of a description of (a) participants’ experience; (b) participants’ perceptions; (c) types of learning; (d) the concept, activities and practices of medical students functioning as module co-directors; (e) commitment factors acting as brakes or levers; and (f) a design guidance. It can also be analyzed in the form of an evaluation of (a) participants’ perception; (b) effects on participants; c) the process; and (d) participants’ commitment factors (see Table 5).

### Research framework

Results show most studies are based on a conceptual framework for analysis. Some are driven by theories from the psychology of learning (a constructivist paradigm for Ha and Pepin [37], a self-regulated learning framework for [62] and [67, 72], an ‘inductive’ approach for [6] and [74], a thematic analysis approach for [52] and [58]), or design science research (a Theoretical Framework of Acceptability (TFA) of healthcare interventions for Brook et al. [85, 15],an evidence-guided redesign approach for Bradshaw et al. [14],the Promoting Action on Research Implementation in Health Services (PARIHS) framework for Ljungblad et al. [60]. However, seven articles do not clearly present their research framework.

### Population

Results show the majority of studies (*n* = 11) include all co-designers in data collection [6, 15, 25, 37, 39, 58, 67, 72, 74]. The study by Ha and Pepin [37] includes the principal investigator in the focus group as a participant. The same is true of Brook et al. [15], who include facilitators in the study population. Eveillard et al. [29], Kayser et al. [52], Kenwright et al. [54] and MacKenzie et al. [62] interviewed or submitted a questionnaire only to students, not to the instructor.

Martens et al. [66] only explored course coordinators’ perceptions (i.e., not students’). Bradshaw et al. [14] distributed their questionnaires to students who had benefited from co-design with a view to highlighting shortcomings in the curriculum, thereby highlighting a need for redesign. Chamunyonga et al. [18] collect data from students involved in co-design to evaluate the product. Similarly, Scott et al. [84] survey both students and faculty members on the use and acceptability of the Ed Reps program among key stakeholders. Tavernier and Wolfe [85] extend their invitation to all current students and faculty, including those not on the Student Faculty Committee, to participate in their study [92].

### Data collection and analysis

Results show nine studies used a *mixed method* including quantitative and qualitative data [15, 18, 25, 29, 52, 54, 67, 74, 84]. Nine other studies collect only *qualitative* data [6, 37, 39, 58, 60, 62, 66, 71, 85] and only one relies on *quantitative* data [14].

Qualitative data are obtained through (a) dedicated research tools such as questionnaires with open-ended comments [18, 25, 29, 52, 54, 62, 67, 72, 74, 84, 85], focus groups [6, 29, 37, 58, 72, 74], interviews [15, 52, 65, 72] ,(b) traces and materials produced as part of the co-design process in five studies (co-construction meetings for Ha and Pepin [37], audio and video of the co-creation workshops for Ljungblad et al. [60], group discussions and students’ usage and their interaction with contents on the online knowledge delivery platform for Kenwright et al. [54], post-its, outputs from the sub-groups, participatory redesign meeting, individual meetings for Harrison et al. [39], focus group meetings for Chamunyonga et al. [18]), and (c) field notes from various stakeholders [15, 37, 39, 62]. Studies carried out an inductive, often thematic, analysis of this qualitative data.

The quantitative data came from questionnaires using a Likert scale. Two studies mobilized questionnaires co-designed by students and faculty [14, 54]. The study by Brook et al. [15] specifies these questionnaires were distributed at different times (before, during and after) in order to explore changes in participants’ opinions during the co-design process. The data was analyzed using Statistical Package for the Social Sciences (SPSS) in four studies [15, 54, 67, 74]. Three studies did not document quantitative data analysis [14, 18, 84].

Table 5 summarizes the characteristics of the studies analyzed.

### Key findings

The outcomes of the 20 studies mainly provide information on the success factors of co-design and its effects on participants (at collective and individual level) and in terms of training transformation.

#### Co-design success factors

**Fig. 2:**
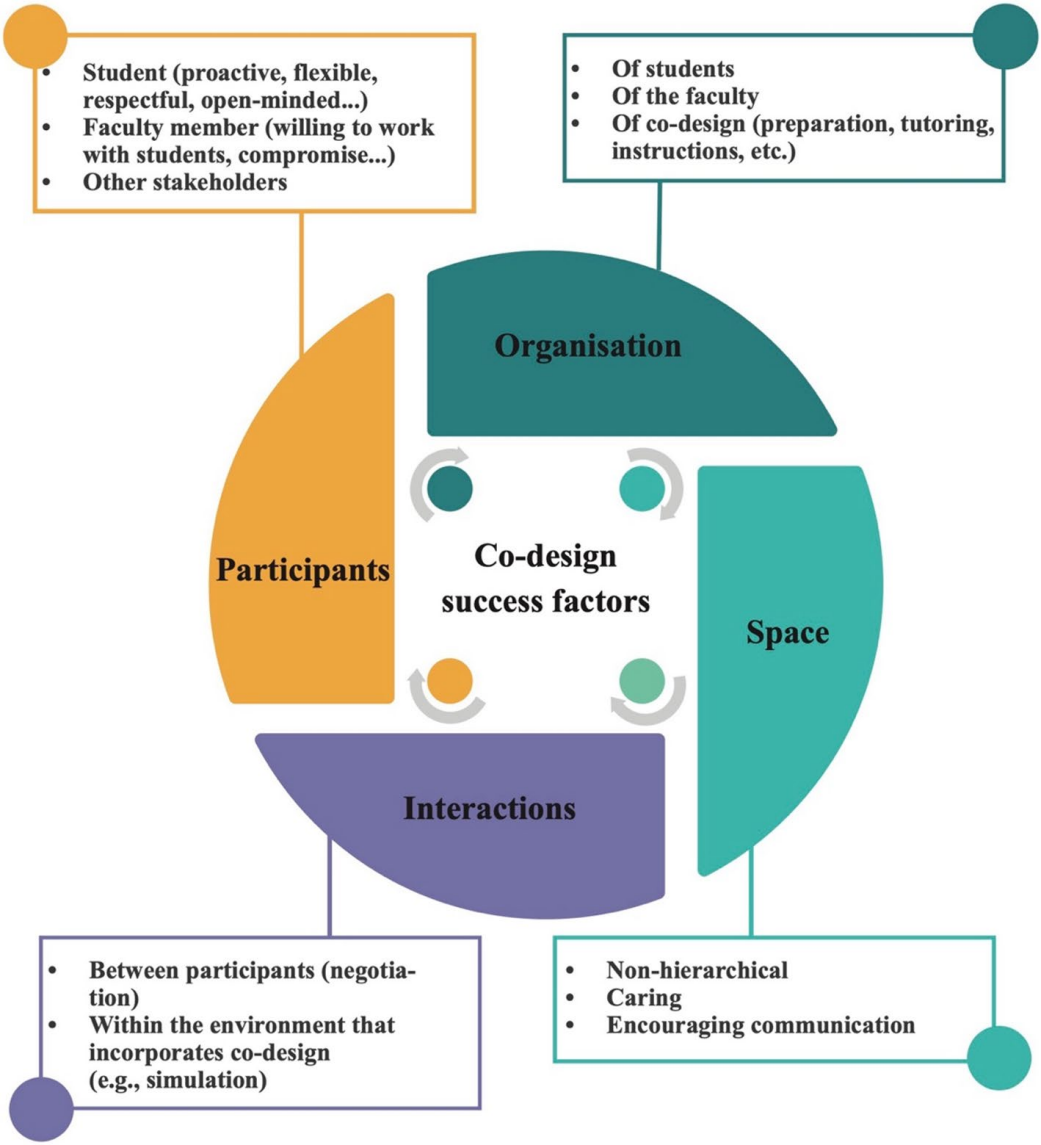
Co-design success factors identified in the studies

Ten studies identified various success factors that facilitate the co-design process. Figure 2 summarizes the findings from these studies.

*Participants*. Several studies highlight key participant attributes essential for a successful co-design process. Meeuwissen et al. [72] identify five specific traits required for effective student-staff collaboration in curriculum co-design: a) attitudes: Students should be proactive, while staff should be open to working with students,b) perspectives: Students should be open-minded, and staff should focus on continuously improving education; c) characteristics: Students should be self-confident, while staff should be familiar with student organizations; d) work ethos: Students should come prepared, while staff should demonstrate dedication to education; e) experience: Students should have familiarity with the educational organization, while staff should have experience as student representatives. Another attribute highlighted in the study by Cosker et al. [25] concerns the presence of young tutors, which was perceived by students as facilitating the development of tutor–student relationships, thereby contributing to a more engaging and collaborative learning environment. MacKenzie et al. [62] add that instructors must be collaborative, responsive, practice-aware, and design-competent. Similarly, students should be collaborative, self-directed, reflective, and aware.

Moreover, Behrend et al. [6] and Laugaland et al. [58] stress that students'motivation for the co-design topic is a major element of co-design success, fostered by the degree of autonomy granted to participants, the enjoyment of group work, the pride students feel in their project, and the satisfaction generated by the inspiration it sparks in others.

*Organization.* The organization of both student representatives and faculty plays a key role in co-design success. Meeuwissen et al. [72] distinguish four features required for optimal student-staff partnership: a) strong internal organization: Student representatives should have structured processes, while the school should foster a culture of appreciation and reward,b) support: Student representatives must build peer support, while the school should provide facilitation; c) professionalism: Student representatives should ensure structured turnovers, while the school should offer training opportunities; d) need for improvement: Student representatives should maintain communication with the student body, while the school should provide coaching, feedback, and evaluation.

A solid organization as a support entity (organization of students and faculty staff) is necessary, but also in the thinking to be developed in the co-design process implementation and the conduct to be adopted (e.g., preparation, instructions, and tutoring). Studies such as Behrend et al. [6], Ha and Pepin [37] and Laugaland et al. [58] highlight that participants emphasized the importance of pre-organization and the structured nature of the workshop, particularly appreciating the provision of a clear agenda and reflective questions in advance.

Martens et al. stress that successful co-design requires organizational support, teacher awareness of student involvement possibilities, and clear communication channels. However, teachers also argue that students may prioritize personal interests over professional relevance and should not bear final responsibility for training choices [66].

In addition, Tavernier and Wolfe [85] highlight several factors perceived as barriers to participation in the Student-Faculty Committee (SFC). While many participants reported no barriers, some cited time constraints and competing priorities. Others expressed a lack of awareness regarding the SFC and its role. Although few indicated that fear of retaliation was a barrier, the authors believe it is an important issue that future SFC activities should address.

*Space.* Establishing an appropriate space for co-design is crucial, particularly in fostering collaboration and engagement among participants. A recurring theme in the literature is the importance of creating an environment where hierarchical structures can be suspended, enabling more equitable participation. Behrend et al. [6] and Laugaland et al1. [58] highlight that hierarchy-free spaces enhance student engagement and encourage more active involvement in the learning process. Behrend et al. [6] demonstrate that joint academic team-building activities, such as presenting at conferences, can mitigate status differences among participants, promoting a more horizontal and inclusive dynamic.

Beyond the physical and structural aspects of co-design spaces, Ha and Pepin [37] emphasize the significance of assembling the right participants to optimize decision-making efficiency. They argue that preparatory steps, including careful selection of group composition, play a crucial role in ensuring productive collaboration: “Gathering the ‘right’ members in the same space enabled the team to make decisions quickly” (p. 93). Similarly, Eveillard et al. [29] report that students perceive the instructor's role as pivotal in maintaining a flexible structure throughout the course. Students appreciated the autonomy granted to them while valuing the instructor’s availability for support when needed. Moreover, they noted that the perception of a weakened hierarchy, in which they were regarded as peers by the instructor, was particularly salient during co-creation activities. This shift in perceived authority contributed to a more collaborative and participatory learning environment.

*Interactions.* Effective co-design relies on well-defined interactions among participants and their environment. MacKenzie et al. [62] describe"critical interaction features"that link students, instructors, and simulations. For instance, in case content negotiation between instructors and students, the instructor calibrates the simulation and acts as a resource when needed. As content experts, they ensure the simulation meets learning requirements while also possessing design skills to support students in their design activities. Students, in turn, are responsible for preparing their roles as patients, observers, or therapists.

The facilitator plays a crucial role in defining tasks and expectations [6], managing time and progress [58], and fostering a low-hierarchy team atmosphere [37].

Additionally, Milles et al. [74] describe two major challenges for student module directors: navigating institutional hierarchy and adapting to the negotiation process in curriculum design, both of which can be sources of difficulty and discouragement.

#### Perceived effects of co-design on training

**Fig. 3:**
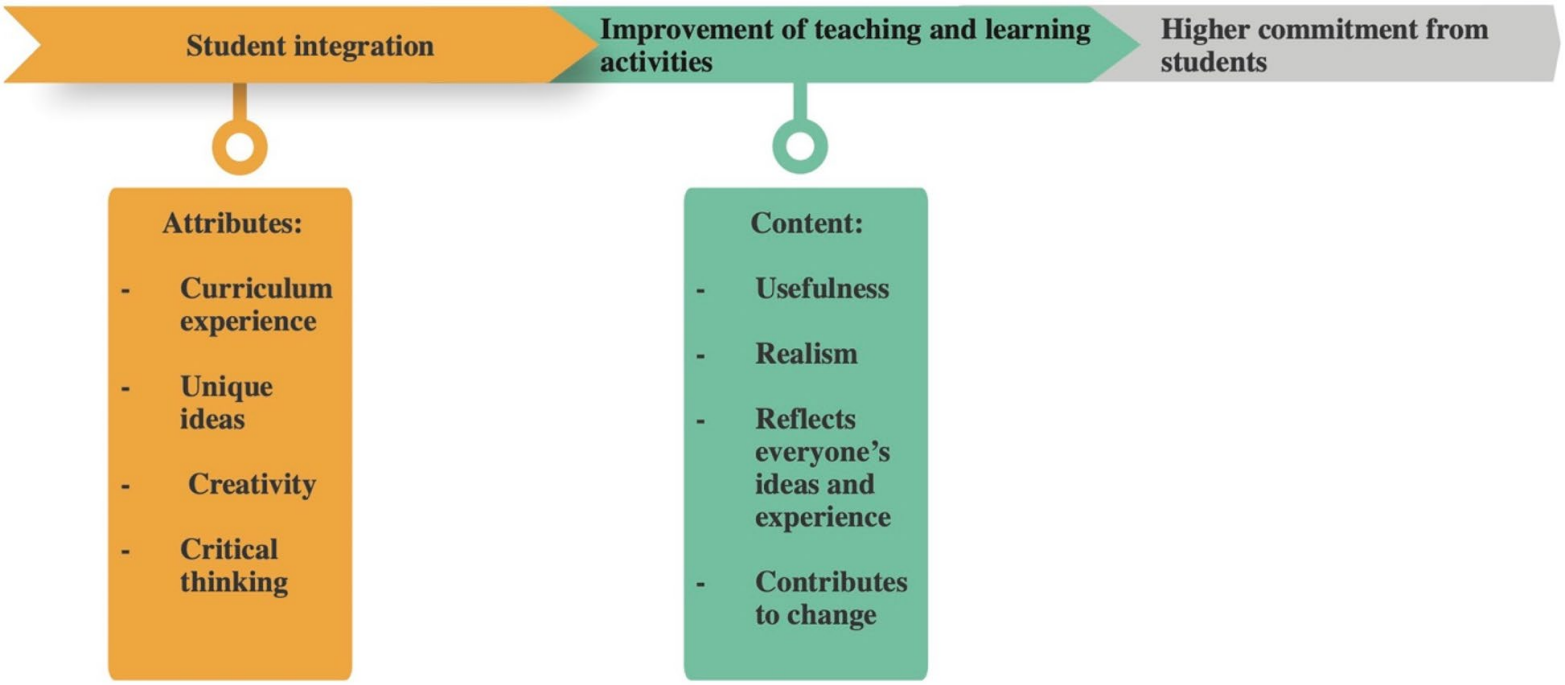
Perceived effects of co-design on training as identified in the studies

In ten studies, participants mentioned some positive effects of co-design on training (Fig. 3).

The involvement of students in the co-design process of teaching activities has positive effects on the quality of training and learner engagement. Martens et al. [66], show that trainers view this collaboration as an opportunity to enhance course content. Due to their experience, students can make unique contributions and propose new ideas, thus enriching the curriculum with useful and realistic content.

Milles et al. [74] also emphasize that student module co-directors are often the only ones capable of having a comprehensive overview of the course content because of their in-depth knowledge of the curriculum. Faculty module directors recognize students'creativity and constructive criticism, although they sometimes note their idealism in light of organizational and budgetary constraints.

The benefits of co-design extend beyond content improvement. According to Brook et al. [15], the strength of this approach lies in the integrity of the final product, which emerges from collective ideas. Ha and Pepin [37] add that the co-created activities have an educational impact both in the short and long term, benefiting students and the program alike. In a study by Cosker et al. [25], a tutor noted that “favorable discussions with these young students… help me to create cases in my specialty (neuro-anatomy)” (p. 1810), highlighting how student interactions can enhance the development of relevant teaching materials.

Student feedback in the study by Kenwright et al. [54] reveals strong emotional engagement with a"student-driven, instructor delivery"learning model and well-structured revision resources. Furthermore, Scott et al. [84] show that most students and faculty agree that the involvement of student representatives positively impacts the curriculum, a finding supported by Milles et al. [74] and Tavernier and Wolfe [85], who note that real-time feedback led to improvements in teaching and learning activities.

Research by Eveillard et al. [29] indicates that students reported a high level of commitment and enjoyment in participating in this new format, finding it more aligned with real-life professional situations they have encountered. Notably, students still remembered the cases they had elaborated and solved four months later and had the opportunity to transfer their learning during their part-time activities in community pharmacies within the few months following the course. Laugaland et al. [58] further add that co-creation workshops were perceived as enjoyable, useful, and instructive, allowing students to share their experiences, gain insights into others'perspectives, and feel that they have a valuable voice.

In conclusion, by integrating students into the design of teaching activities, educational institutions can not only enhance the quality of content but also strengthen student engagement, thereby creating a rich and relevant learning experience.

#### Effects of co-design on collaboration patterns

**Fig. 4:**
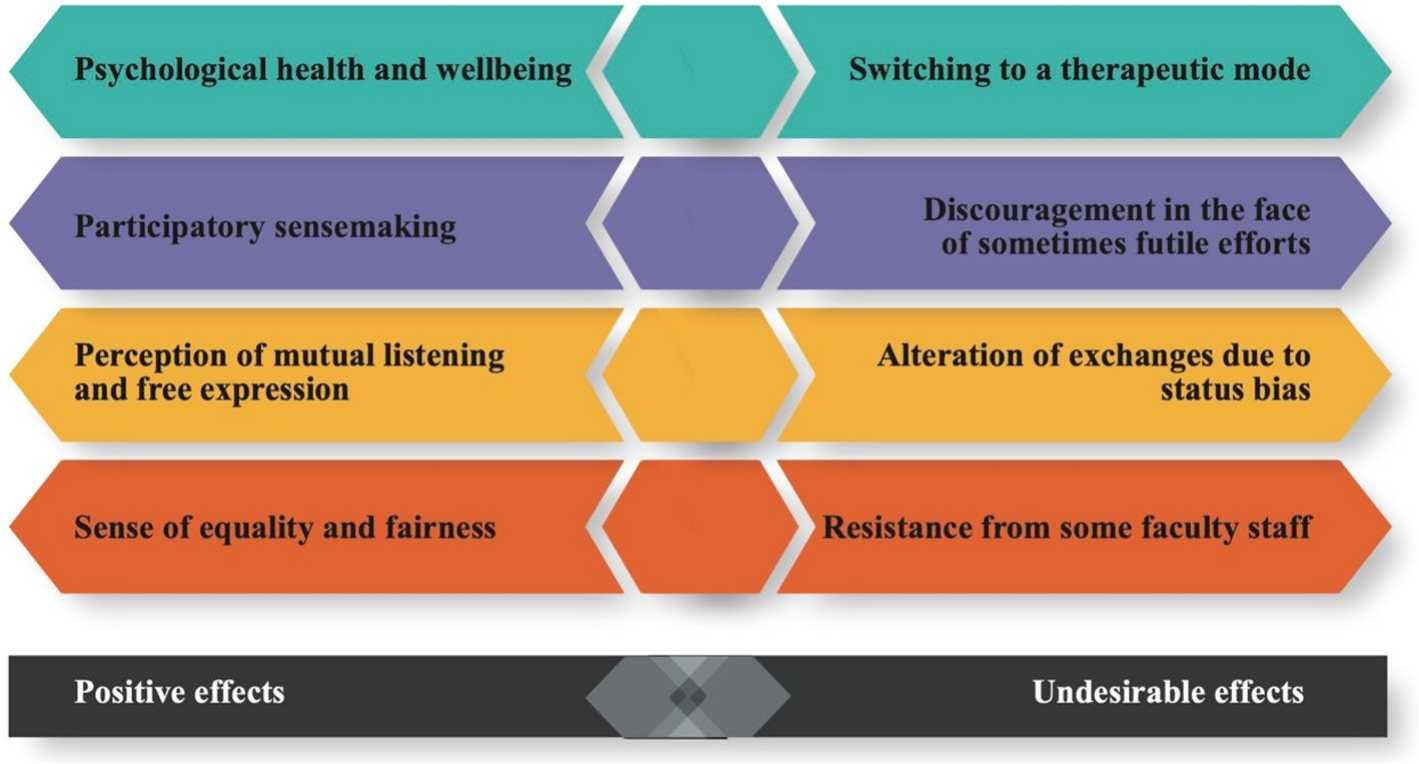
Effects of co-design on collaboration modes as uncovered in the studies

Engaging participants as equal partners in co-design significantly influences their collaborative experience. While research underscores its benefits, it also identifies challenges that must be addressed in structuring such initiatives. Figure 4 synthesizes key findings from previous studies, illustrating both the advantages and potential drawbacks of co-design practices.

One major benefit is its impact on psychological health and well-being. Co-design fosters a supportive environment where participants feel heard and reassured, as shown by Brook et al. [15]. Their study describes how the ability to share clinical placement experiences and validate emotions contributed to a sense of mutual support. This aligns with the salutogenic model, which emphasizes the preservation of well-being through positive social interactions. However, as highlighted in the figure, there is a risk of switching to a therapeutic mode if the focus drifts too much towards emotional support rather than productive collaboration.

Another key advantage is participatory sensemaking, where participants engage in meaningful discussions, shaping their collective understanding of the co-design process. Ha and Pepin [37] emphasize the value of these interactions, where every participant feels their contributions matter a point also highlighted by Kayser et al. [52]. Behrend et al. [6] state faculty members value the collaboration they have with students because it gives them access to their points of view, thereby enabling them to test their project’s feasibility. For trainers, working collaboratively with students in the team provided new arguments for why these projects make sense. Nonetheless, despite this engagement, some experience discouragement in the face of sometimes futile efforts. Studies by Kenwright et al. [54] and Meeuwissen et al. [72] reveal that more than half of student representatives feel their input does not translate into actual change, leading to frustration and disengagement.

Co-design also enhances the perception of mutual listening and free expression. A hierarchy-free space allows participants to voice their opinions openly, as noted by Brook et al. [15] and Ha and Pepin [37]. Scott et al. [84] describe how student representatives act as mediators between students and faculty, facilitating communication. Yet, Harrison et al. [39] and Laugaland et al. [58] warn that, in practice, exchanges can be altered due to status bias. Subtle power dynamics may limit the extent to which students influence discussions, even when they actively contribute. They propose separate workshops to counteract these imbalances, fostering more open and critical discussions.

Finally, co-design fosters a sense of equality and fairness, as faculty members increasingly recognize students as essential stakeholders in the process. In the study by Ha and Pepin [37], participants describe their collaboration during co-design as an original and unique experience marked by a profound sense of unity where “every member felt they had a worthwhile contribution to the team’s work” (p. 93). This result aligns with Eveillard et al. [29], who found that students felt their teachers regarded them as peers within a partnership perspective. This temporary"peer status"could explain why students in their study did not wish to be evaluated during team-based learning, as such evaluation might have altered the nature of the partnership and their perception of equality in the classroom. However, despite these positive aspects, resistance from some faculty staff remains. Some educators hesitate to integrate students into decision-making processes, either due to institutional constraints or personal reluctance [74]. This resistance can slow down decision-making and create tensions within co-design teams.

In conclusion, while co-design fosters well-being, participation, mutual listening, and equality, it also presents risks related to therapeutic shifts, discouragement, status bias, and faculty resistance. A well-structured approach—including clear guidelines, facilitator training, and adaptive strategies—can help maximize its benefits while minimizing these undesirable effects.

#### Effects of co-design on individuals

Nine studies highlight the significant impact of the co-design process on participants, particularly students. Behrend et al. [6] demonstrate that co-design fosters students'professional development by enhancing their knowledge of inter-professionality through close collaboration with participants from diverse professions and statuses. This collaborative experience not only shifts students'attitudes towards interprofessional collaboration but also cultivates essential skills such as self-confidence [54], courage to speak up, and communicative interprofessional competencies. Additionally, students develop academic and teaching-related skills, including the ability to deliver presentations at conferences, an aspect corroborated by Kayser et al. [52] and Meeuwissen et al. [72]. Meuuwissen et al. [72] reveal that student participation in governance provides valuable learning and career opportunities, immersing them in professional decision-making within complex organizations. As a result, students acquire a broad spectrum of skills, such as communication, strategic and metacognitive thinking, argumentation, debating, networking, lobbying, leadership, and organizing. They also strengthen their ability to collaborate with peers and faculty staff while deepening their understanding of organizational dynamics. However, the study highlights a challenge: 48% of student representatives report a lack of support from staff members, including insufficient feedback, coaching, or response to actions, which ultimately leads to demotivation.

Beyond governance, co-design cultivates transferable skills across various learning contexts. Brook et al. [15] illustrate how both group members and facilitators benefit from this process. Students develop problem-solving abilities, creative thinking, knowledge sharing, and consensus-building skills to find solutions, alongside enhanced communication. Facilitators, in turn, gain confidence and competence in supporting group work. Similarly, Ha and Pepin [37] highlight the transformative effect of co-design on students'perspectives on learning and teaching, fostering curiosity and deeper engagement. Educators also experience a shift, gaining awareness of the impact their teaching decisions have on students'workload. Through self-reflection, all participants exhibit increased motivation and involvement.

The effects of co-design extend to simulation-based learning. MacKenzie et al. [62] demonstrate that co-constructed simulations help students build confidence, fostering independent self-regulated learning essential for clinical practice. This adaptability aligns with Martin et al. [67], whose study confirms participants'general satisfaction with co-constructed simulations, further emphasizing their practical benefits.

While these studies primarily focus on students, the influence of co-design on faculty staff and facilitators remains less explored. Nevertheless, Harrison et al. [39] provide an important perspective on the role of prior assessment experiences in shaping collaborative redesign efforts. They argue that prior experiences act as a filter, influencing discussions and potentially restricting the scope of proposed changes. This highlights a critical factor to consider in co-design processes, as participants'previous experiences can impact group dynamics and the breadth of innovation.

## Discussion

Our synthesis and qualitative analysis of studies on students as co-designers in health professional education clarified this emerging research and practice field. The 20 included studies demonstrated narrow scopes, varying sample sizes, and generally lacked detailed frameworks, methods, and results, largely due to diverse educational contexts and professional fields. This variability complicates quality assessment and synthesis, constituting the main limitation of our work. Nevertheless, this review provides an accurate overview of recent developments.

Three key discussion areas emerge: first, recognizing co-design as an emerging practice needing structured guidance; second, exploring the potential of Change Labs as theory-driven co-design support; and third, developing co-design as a rigorous research domain.

### Co-Design as an emerging practice requiring guidance

Various terms describe co-design (e.g., co-creation, students as partners, student engagement). Although student-staff partnership is most common, the distinctions among terms remain unclear. The absence of a consensus definition complicates research and practice. Clarifying the level of student involvement is essential. Our review included only studies in which students significantly participated in structured co-design activities, leading to concrete educational changes (e.g., course content, curricula).

Results show co-design typically aims at improving content authenticity and student engagement. This occurs when students actively shape training content aligned with their needs and real-world practices. Often, however, educators rely on unexamined assumptions, expert opinions, or scientific literature, creating tension between retaining and delegating decision-making power [66]. The literature emphasizes respect, power negotiation, and shared responsibility.

Co-design serves five primary purposes: (a) course development, (b) simulation creation, (c) assessment, (d) program design, and (e) educational interventions. Studies typically describe project phases but neglect detailed task descriptions for stakeholders. Training topics are often arbitrarily chosen or recommended by external authorities, resulting in fragmented and generic guidelines.

Despite our analysis, clear guidelines for implementing co-design in specific contexts (e.g., simulations, assessments) remain elusive. Recommendations often focus on participant attributes rather than practical co-design strategies. The role of students in identifying training needs and methods for structuring their involvement are inadequately defined. Concrete guidance for facilitating stakeholder interactions, reflective dialogue, and collaborative practice development is lacking.

To improve, co-design should become structured, theory-driven, and practically effective, translating student participation into meaningful, sustainable training improvements. Recommendations include: (i) differentiated frameworks for co-design purposes; (ii) clear criteria for context-specific implementation; (iii) involving students early in identifying training gaps; (iv) structured methodologies for integrating student insights; (v) focusing on practical strategies rather than personal attributes; (vi) guidance on balancing contributions and mitigating hierarchies; (vii) structured collaboration tools for stakeholder interaction; and (viii) boundary objects to align diverse perspectives (e.g.,"Change Labs").

### “Change Labs” as a promising theory-driven way to support co-design practices

Involving students in training design aims at creating content that is authentic, relevant, and engaging. Recent literature emphasizes co-design’s potential to enhance student engagement across cognitive, affective, behavioral, agentic, and socio-cultural dimensions [51]. Yet, student perspectives are often neglected [18, 27], "Change Laboratory interventions"offer useful resources (e.g., [38, 88]). They encourage multi-voiced dialogue, combining experiential reflection and intellectual analysis through"mirror materials"(e.g., videotaped practices, critical incidents). This method prioritizes real-world practices, fostering transformative agency among participants, considering the main challenge in co-design sessions is to set up an environment and resources allowing participants to activate their capacity to break away from the given frame and to act proactively to transform it collaboratively.

Change Labs exemplify systematic integration of objectives (co-design), theory (activity and learning theories), and methodology (collaborative design). Such structured, theory-driven frameworks could significantly enhance co-design initiatives in health professional education.

### Structuring Co-Design as a Research Field

Co-design in health professional education remains underdeveloped, lacking empirical evidence and rigorous methodologies. Existing studies predominantly focus on success factors, neglecting systematic examination of learning outcomes, training effectiveness, or learning processes in co-design contexts. Most reported benefits are anecdotal, relying on self-reports about student creativity and input.

Students reportedly experience professional growth, increased confidence, motivation, and self-regulated learning through co-design. Yet, research has not thoroughly investigated knowledge acquisition or learning processes. Malet et al. [64] highlight the potential of integrating co-design into simulation-based training, emphasizing the need for robust methodologies and theoretical grounding. Current research lacks controlled experimental or rigorous qualitative designs.

Advancing the field requires rigorous research designs isolating co-design effects. Promising protocols exist [81, 87] but require implementation. Future research should clarify distinctions between co-design and broader collaborative methods, using robust qualitative approaches (interaction analysis, phenomenological studies) to explore participant interactions, learning processes, and subjective experiences. Co-design in health professional education thus remains a rich area for future research.

## Conclusion

Our literature review identifies some key insights on student-staff partnerships in health professional education. Some align with recent findings in related higher education literature, while others offer original perspectives.

Research on student-staff partnerships has notably increased since 2010, as shown in studies such as Bovill [11], yet its adoption in health professional education remains limited [5]. Existing studies often emphasize role dynamics and interpersonal relationships rather than explicitly exploring co-design as a structured practice. Consequently, co-design is emerging but lacks clear methodological guidance within health education research.

A significant conceptual challenge is that terms like co-design, co-creation, co-construction, and participatory design are often used interchangeably, creating confusion despite reflecting specific disciplinary and cultural. Although some studies employ established theoretical models—such as Participatory Action Research, Participatory Design Principles, Students as Partners, or Students as Module Co-Directors—many lack a clearly defined theoretical foundation.

Most documented co-design programs are pilot initiatives or short-term projects, resulting in limited evidence concerning their long-term implementation and sustainability. Additionally, power asymmetry between faculty and students remains a critical concern, leading some authors to advocate for increased student representation within co-design teams.

Future research should address several important areas: establishing clearer definitions and robust theoretical frameworks, exploring long-term impacts and sustainability of co-design initiatives, and tackling persistent challenges related to student participation and effective collaboration with faculty.

Co-design in health professional education is an emerging but underdeveloped research area. While there are clear benefits, the lack of standardized frameworks, long-term studies, and institutional support limits its widespread implementation. Further research is needed to provide structured guidance and empirical validation of co-design practices.

## Supplementary Information

Supplementary Material 1.

## Data Availability

Data sharing is not applicable to this article as no new datasets were generated or analyzed during the study. All databases used in this study are publicly available. Queries can be found in the appendix.
