# Supplementary material for: Students as co-designers in health professional education: a scoping review
Source: BMC Med Educ. 2025 May 3;25:645. doi: 10.1186/s12909-025-07110-0 (PMC12049780; doi:10.1186/s12909-025-07110-0)
Supplement: Supplementary file 1 — Supplementary Material 1. [file 12909_2025_7110_MOESM1_ESM.docx]

**Additional files**

**Table A1 Queries used for performing the search process in each online database**

| **Online databases** | **Search query** |
| --- | --- |
| Cochrane Library | *Q1*"participatory design" OR "co**-**design" OR "co**-**creation" OR "co**-**construction" OR "co**-**production" OR "co**-**development" OR "collaborative design" in Title Abstract Keyword AND "learner" OR "student" OR "trainee" in Title Abstract Keyword - with Publication Year from 2010 to 2023  *Q2* "student staff partnership" OR "student staff collaboration" OR "student faculty collaboration" OR "student faculty partnership" OR "students as partners" in Title Abstract Keyword - with Publication Year from 2010 to 2023  *Title – abstract – keywords* |
| Ovid | *Q1* (("participatory design" or "co-design" or "co-creation" or "co-construction" or "co-production" or "co-development" or "collaborative design") and ("student" or "students" or "learner" or "learners" or "trainee" or "trainees")).ti.kw.ab.*Q2* ("student staff partnership" or "student staff collaboration" or "student faculty collaboration" or "student faculty partnership" or "students as partners").ti.kw.ab. *Title – Abstracts – Keywords* |
| PubMed | *Q1* (participatory design[Title/Abstract] OR co design[Title/Abstract] OR co creation[Title/Abstract] OR co construction[Title/Abstract] OR co production[Title/Abstract] OR co development[Title/Abstract] OR collaborative design[Title/Abstract]) AND (student[Title/Abstract] OR students[Title/Abstract] OR learner[Title/Abstract] OR learners[Title/Abstract] OR trainee[Title/Abstract] OR trainees[Title/Abstract]) Filters: Classical Article, English, from 2010 – 2023  *Q2* (student staff partnership[Title/Abstract] OR student staff collaboration[Title/Abstract] OR student faculty collaboration[Title/Abstract] OR student faculty partnership[Title/Abstract] OR students as partners[Title/Abstract]) Filters: Classical Article, English, from 2010 - 2023  *Title – abstract* |
| Science  Direct | *Q1* TITLE-ABSTR-KEY ("participatory design" OR "co-design" OR "co-creation" OR "co-construction" OR "co-production" OR "co-development" OR "collaborative design") AND ("student" OR "learner" OR "trainee")  *Q2* TITLE-ABSTR-KEY ("student staff partnership" OR "student staff collaboration" OR "student faculty collaboration" OR "student faculty partnership" OR "students as partners")  *Title – abstract – keywords* |
| Scopus | *Q1* TITLE-ABS-KEY ( ( "participatory design" OR "co-design" OR "co-creation" OR "co-construction" OR "co-production" OR "co-development" OR "collaborative design") AND ( "learner" OR "student" OR "trainee" ) ) AND ( LIMIT-TO ( DOCTYPE , "ar" ) ) AND ( LIMIT-TO ( SUBJAREA , "MEDI" ) OR LIMIT-TO ( SUBJAREA , "HEAL" ) OR LIMIT-TO ( SUBJAREA , "NURS" ) ) AND (LIMIT-TO ( PUBYEAR , 2023 ) OR LIMIT-TO ( PUBYEAR , 2022 ) OR LIMIT-TO ( PUBYEAR , 2021 ) OR LIMIT-TO ( PUBYEAR , 2020 ) OR LIMIT-TO ( PUBYEAR , 2019 ) OR LIMIT-TO ( PUBYEAR , 2018 ) OR LIMIT-TO ( PUBYEAR , 2017 ) OR LIMIT-TO ( PUBYEAR , 2016 ) OR LIMIT-TO ( PUBYEAR , 2015 ) OR LIMIT-TO ( PUBYEAR , 2014 ) OR LIMIT-TO ( PUBYEAR , 2013 ) OR LIMIT-TO ( PUBYEAR , 2012 ) OR LIMIT-TO ( PUBYEAR , 2011 ) OR LIMIT-TO ( PUBYEAR , 2010 ) ) AND ( LIMIT-TO ( LANGUAGE , "English" ) )  *Q2* TITLE-ABS-KEY ("student staff partnership" OR "student staff collaboration" OR "student faculty collaboration" OR "student faculty partnership" OR "students as partners") AND ( LIMIT-TO ( DOCTYPE , "ar" ) ) AND ( LIMIT-TO ( SUBJAREA , "MEDI" ) OR LIMIT-TO ( SUBJAREA , "HEAL" ) OR LIMIT-TO ( SUBJAREA , "NURS" ) ) AND (LIMIT-TO ( PUBYEAR , 2023 ) OR LIMIT-TO ( PUBYEAR , 2022 ) OR LIMIT-TO ( PUBYEAR , 2021 ) OR LIMIT-TO ( PUBYEAR , 2020 ) OR LIMIT-TO ( PUBYEAR , 2019 ) OR LIMIT-TO ( PUBYEAR , 2018 ) OR LIMIT-TO ( PUBYEAR , 2017 ) OR LIMIT-TO ( PUBYEAR , 2016 ) OR LIMIT-TO ( PUBYEAR , 2015 ) OR LIMIT-TO ( PUBYEAR , 2014 ) OR LIMIT-TO ( PUBYEAR , 2013 ) OR LIMIT-TO ( PUBYEAR , 2012 ) OR LIMIT-TO ( PUBYEAR , 2011 ) OR LIMIT-TO ( PUBYEAR , 2010 ) ) AND ( LIMIT-TO ( LANGUAGE , "English" ) )  *Title – abstract – keywords* |

**Table A2 Summary of the co-design process in studies (NS=not specified)**

|  | **Temporality** | **Content and Organization** | **Task for the co-design phase** |
| --- | --- | --- | --- |
| Behrend et al., (2019) | NS | Two interprofessional education projects:  1. Both of the projects were structured into a steering group and a working group (supported by a project coordinator). The same students were members of the steering and working groups  2. The students’ work results were regularly reported to the steering group, which reviewed proposals, gave feedback and made decisions | 1. The steering groups met regularly and made strategic decisions, defined milestones, and reviewed project progress.  2. The working groups acted in an operative manner (development of specific course content, teaching and learning materials, evaluation forms, implementation of the tutorials in the project) |
| Bradshaw et al., (2017) | NS | Based on the Kern model of medical curricular development:  Phase 1: Problem identification and general need  Phase 2: Targeted needs assessment  Phase 3: Goals and objectives  Phase 4: Educational strategies  Phase 5: Implementation  Phase 6: Evaluation and Feed-back | 1. The working group (comprised of faculty and students) developed a fourth- year, month long elective rotation and a revised reading list  *Detailed description of courses in table format*  The tasks were to allow the students to examine the way in which their curriculum taught pain and to produce a new curriculum map.  2. Two other students enrolled in the elective made curriculum refinements  3. One of them (with Faculty) implemented the curricular changes proposed, co-designed a 30-minute didactic session, co-designed a survey assessing knowledge and attitudes toward pain |
| Brook et al., (2020) | 6 meetings over 3 months | Guided by the established co-production toolkit, Experience Based Co-design (EBCD) in 10 steps presented in a table:  - Material: Literature and creation of a film outlining the issues and evidence base  - Six meetings facilitated by early career nurse and research fellow  - Organization of an early career nurse feedback event  - Organization of a student feedback event  - Organization of a joint student-early career nurse event | Discussion of areas to focus on for intervention development |
| Chamunyonga et al. (2018) | 1-hour 4 weekly meetings | 1. Four semi-structured qualitative focus-group discussion with third year cohort  2. Implementation, survey(s), outcomes, impact with second year cohort | 1. First meeting: the university coordinator providing students with key information on the value of partnership approaches in pedagogical transformations. Students nominating two alternative assessment approaches for a treatment planning subject and providing a brief description of each task (authenticity, realism, advantages, disadvantages)  2. Three other meetings: identify benefits, challenge and discuss how to implement the tasks. Two tasks were chosen |
| Cosker et al., 2021 | Phase 1: 4h  Phase 2: NS  Phase 3: 4 circuits (1 circuit = 5 stations = 110 min per student) scheduled for 7 half-days.  Phase 4: 25-min | Phase 1: training sessions, which were organized separately for tutors and students by the members of the OSCE teaching staff  Phase 2: development and rehearsal of OSCE stations by tutor-guided groups of five students  Phase 3: practice OSCE sessions with post-encounter feedback by tutors  Phase 4: final debriefing of the OSCE station | NS |
| Eveillard et al., 2021 | NS | 1. Each group was divided into six self-constituted subgroups of three or four students.  2. The role of the teacher was to help the students consider the main issues related with the case in terms of diagnosis, therapeutics and prevention, while giving them great latitude in developing the scenario. | Each subgroup **created a clinical case** on a basis of little ill-structured contextual information (vignettes), which were provided by the teacher. Cases were co-created during the first in-class session (TS1) by the way of interactions between the teacher and each group of students. These interactions occurred at least three times during this first session, allowing student/teacher co-creation. |
| Greenhouse et al., 2022 | NS | NS | NS |
| Ha & Pepin (2017) | 1. 3 x 2 hours Meetings  2. September  2014 to December 2014 | Three phases:  1. Preparing co-construction: preparatory decision to guide teamwork  2. During co-construction (facilitated by the Principal Investigator): together for the first two meetings, in two groups for each activity, then all together again  3. After co-construction (led by a moderator): focus group discussion (included IP facilitators) | Three meetings:  1. First meeting: Introduction, working definition of Clinical Nursing Leadership (CNL), brainstorming yielded 17 possible learning activities  2. Second meeting: review of the first meeting and members’ suggestions (contextualisation with the research setting’s competencies framework and a definition)  Reducing the list of possible learning activities to two  3. 3^rd^ meeting: details about both activities |
| Harrison et al., (2017) | A 2h30 joint meeting  A 30-minute interview within 1 week of the participatory design meeting | 1. Based on the Combination-Of-Perspectives (COOP) model  2. Co-design with thoughtful group organization (single, mixed, all together) followed by individual follow-up interviews | 1. A short presentation explaining the problem by the lead researcher (preparation, instructions)  2. A co-design by using blue sky thinking to design solutions for this problem  3. Group members were then encouraged to critically challenge their proposals in order to explore the underlying factors which would support (or hinder) successful practical implementation of the assessment change  4. In the interview, participants were encouraged to consider the personal consequences for them as an individual, if the proposed changes were to be implemented |
| Kayser et al., 2022 | NS | NS | The students were involved in preparing the objectives for the course, preparing pre-course material, including relevant literature and short online video presentations as preparation for the course. |
| Kenwright et al., (2017) | Course revision during 8 weeks | Two project cycles:  1. Needs identification with 2 meetings in small group (first meeting: students, second meeting: student representatives and instructor)  2. Implementation: Dynamic interaction modified the content and delivery of these sessions | Cycle 1: first meeting: students in small group express their opinions about content and method of delivery; second meeting: students and instructors negotiated the feasibility of students’ proposals (thematic analysis) until agreement. Students formulated their own topics, and there were no constraints in topic choice  -Cycle 2: four face-to-face sessions and four online sessions |
| Laugaland et al., 2023 | 2,5-hour separate workshops + one joint 3,5 hour  workshop | The separate workshops aimed to explore stakeholders’ challenges with clinical placements in nursing homes; A joint workshop with RN mentors, student nurses, nurse educators, and e-learning designers was conducted to ideate solutions and provide input into the content and functionality of the resource, including how to accommodate key challenges. | Defining - Ideating - Prototyping share experiences, define problem areas, and ideate solutions; identify areas in need of improvement; and create informational, contextual, and educational content to be included in a digital educational resource. |
| Ljungblad, et al. (2022) | 4 Zoom workshops across six months (70 to 111 minutes) | NS | 1. Several creative tools in the workshops to stimulate participant engagement.  2. To ensure that all participants’ voices were heard, follow-up questions were asked to give each participant the opportunity to contribute.  3. The themes that emerged from the analyses were then translated into a prospective newborn resuscitation course for midwifery students. |
| MacKenzie et al., (2018) | Series of 2 simulations per week from Tuesday to Thursday | 1. Preparation: the first simulation required each student to prepare a task to teach a colleague in 25 min  2. Seven stages (Module creation, Case creation, Simulation preparation, Case pre-brief, Simulation and recording, Individual and group debrief, Reflection and refinement)  3. Students divided into two interchangeable groups:  Group A > patient and observer/debriefer  Group B > therapist | Example of case pre-briefing:  1. Instructor: Collaborate to clarify key features, interventions, and equipment. Refine final co-constructed case  2. Student patient: collaborate and co-construct final case features with refinement by instructor  3. Student observer: as above  4. Student therapist: self-regulated learning preparation of case key features, plan session, and complete identified key skills  *Detailed description of tasks in a table* |
| Martens et al., (2020) | NS | NS | 1. Course coordinators may choose to collaborate with students in their course design teams, by asking their input on aspects of the course that need improvement  2. Maastricht University affords students the opportunity to fill in evaluation questionnaires and to participate in an evaluation panel or course design team |
| Martin et al., (2020) | Each session took 6 hours conducted at 1-month intervals between November 2019 and May 2020 | 1. Preparatory session  2. Six Session of simulation with case preparation and scriptwriting, editing, case clarification, role play, and script finalization with the Standardized Patient (SP) and supervisor, the simulation session itself | 1. A learner, named clinician, create a case base sur a clinical encounter faced during training or clinical practice  2. During the preparation of the case, the learning goals are jointly elaborated and refined by the triad of clinician, supervisor, and actor. It includes a rehearsal with the SP (making the SP role more precise, reflexion on the scenario by the clinician). Next, a fellow-learner (a peer or blinded supervisor, the ‘interviewer’) is provided a ‘door note’ with brief background information of the case, before interviewing the SP |
| Meeuwissen et al., (2019) | NS | NS | 1. In the Netherlands, student representatives are able to participate in governance (quality assurance, management and decision-making)  2. Students are represented at the university council, faculty council or student council  *Described in more detail in the Appendix* |
| Milles et al., (2019) | The students’ work is compensated with a Euro equivalent of 10 working hours per module each month | 1. Each module of the integrated program is led by a group of four module directors: three faculty members from various disciplines, and one medical student.  2. The four module directors are elected by, and also chair, a specific module planning group, consisting of delegates from all disciplines involved in the teaching of that module. They form the module directory board and are jointly responsible for the cyclic curricular development process of their module, e.g. quality assurance and future improvement. | 3 categories of tasks  1) During the module ran (introducing the current cohort to the module, serving as the main contact person for the students)  2) Between the module runs (synthesis evaluation of students,discussion among the module directors about the current module design, student evaluation results, and suggestions for improvement,...)  3) Self-organization of the group by all student module co-directors (Contributing to the meetings with all student module co-directors, Overseeing the selection of new student candidates as module co-directors and introducing them to their tasks …)  *Tasks descriptions in table and appendix* |
| Scott et al., (2019) | Students were selected in first pre-clerkship. and continued their service longitudinally through their clerkship and beyond | 1. The Ed Reps program was launched alongside the new curriculum to foster a partnership culture between faculty and students for continuous and real-time curricular improvement  2. A faculty mentor provides support (participating in the Ed Reps’ biweekly meetings, available to meet with Ed Reps on an ad hoc basis) and training (adult learning theory, the evidence-based teaching strategies, best practices for providing feedback to faculty and peers, and procedures for connecting fellow students to institutional supports as needed) | 1. EdReps met biweekly to review the gathered feedback, identify challenges, and generate solutions.  2. Met regularly with course directors and core faculty to convey bidirectional feedback  3. Updated their class on faculty responses to student feedback via weekly emails and in-person announcements  *Detailed description in table form* (Program description, eligibility and selection, example activities, key differentiating features) |
| Tavernier & Wolfe, 2022 | two years of implementation | 1. Students and faculty developed a continuous quality improvement (CQI) partnership, the Student Faculty Committee (SFC).  2. The SFC meet monthly throughout the academic year  The Plan Do Study Act (PDSA) process was utilized by the student faculty CQI committee.  3. Students are responsible for the recruitment and selection of student participants | 1. Student responsibilities:  collect feedback from the student body; Presentation of recommendations for program improvement to faculty members; collaborate with faculty members for solutions; develop activities and/or programs for student engagement and success; creating meeting agendas, facilitating meetings, recording minutes, and publishing a student newsletter.  2. Faculty members responsibilities:  create and maintain a safe and professional environment; collaborate with student members for solutions to issues; present concerns and recommendations from SFC meetings to the nursing faculty; recommend student centered changes in program policies and procedures; support and facilitate student members with projects for program improvement. |
